# Removal of a Large Fibro-Sarcoma of the Uterus by Abdominal Section under Lister’s Method

**Published:** 1880-02

**Authors:** L. C. Lane

**Affiliations:** Prof. of Surgery Medical College of the Pacific


### Selections.

---

### REMOVAL OF A LARGE FIBRO-SARCOMA OF THE UTERUS BY ABDOMINAL SECTION UNDER LISTER'S METHOD, WITH SUCCESSFUL RESULT.

BY L. C. LANE, M. D., PROF. OF SURGERY MEDICAL COLLEGE OF THE PACIFIC.

In the *Pacific Medical and Surgical Journal* for August, a case of this nature is reported by F. H. Dennis, M. D., of San Francisco.

The patient, the wife of the physician reporting the case, had reached the age of 35; the tumor was first noticed fifteen years previously—the patient then being 20 years of age, and having menstruated regularly about a year. She enjoyed perfect health until within a short time preceding the time of operation. The writer proceeds to say: "As her health was good, no thought of an operation was entertained by any one, but on the contrary all opposed it, notwithstanding which, she anxiously wished to be relieved of the tumor on account of her enormous size and the menorrhagia. About the 15th of April her health began to fail. She commenced to fall away in flesh; her appetite, which had always been remarkably good, became impaired; œdema of the extremities was very extreme, owing to pressure on the femoral veins; altogether she failed very rapidly. The pressure was more on the left disc, the larger portion of the tumor being on that side. Her left leg was simply enormous in size. For several days before the operation she could not walk across the room, and her pain was beyond endurance; gangrene of the leg would have been the inevitable result within a few days." The operation being decided upon, it was performed May 27th, as follows: "The usual preparations were made, such as giving an enema twelve hours before; no breakfast being allowed in the morning. Several gallons of previously boiled water were in readiness, with which to make the carbolized water. One, strength of five per cent. carbolic acid was prepared for the spray, and one of two and one-half per cent. for sponges, ligatures and instruments, and a furnace with heated irons in readiness to be used for cauterizing, if found necessary. The steam spray was made ready with its five per cent. solution, and other necessary preparations having been made, she was brought in and placed on the operating table. Dr. Lane assisted by Drs. J. H. Wythe, Burgess, Sims, Plummer, J. Regensburger, M. Regensburger, Perry, Jos. Haine and myself, commenced the operation. The anæsthetic having been administered, an incision commencing four inches above and to the left of the umbilicus in the linea alba, and extending nine inches down and terminating over the fundus of the bladder, was made under a full spray of carbolic acid solution. On dividing the peritoneum, the tumor a large nodulated, interstitial uterine fibro-sarcoma, came directly into view. The abdominal walls being held apart by broad-bladed retractors in the hands of an assistant, it was then lifted partly out of the cavity. There were found adhesions anteriorly to the fundus of the bladder, and great difficulty was had in detaching or dissecting it away, so closely was it adherent. To avoid puncturing the bladder, a curved catheter was introduced and kept in constant motion, moving it from side to side to act as a guide for the dissection. The tumor was also found adherent laterally and posteriorly, and there likewise was with difficulty detached, owing to the close proximity of the internal iliac artery and vein; the superficies of the attachment being equal to a space ten inches in length by six inches in breadth. The intestines with the omentum were carefully wrapped in Lister's carbolized gauze, and held to one side in order to avoid wounding them, the spray falling all the while in a steady "mist" into the abdominal cavity. A stout curved needle, armed with a strong carbolized double silk ligature, was made to transfix the uterus through the cervix about one inch above the os. Each half was then firmly tied, including the uterine artery and vein, after dividing the ovarian and branches of the uterine artery, in the broad and round ligaments, one-half of the main ligature gave way, and some little difficulty was had in arresting hemorrhage. It was finally, however, held in check with an ovariotomy clamp, until the ligature was again tied lower down. It was very fortunate that the accident occurred when it did, for had it happened after the wound was closed she might have bled to death before it could have been gotten at to tie.

After all the bleeding was arrested, the whole tumor, including the uterus, ovaries and fallopian tubes, was divided and removed *all together.* The end of each (half) ligature was then attached to a silver wire and brought down through the cervix and also into the vagina, thus establishing through the course of the ligature a *drainage;* all oozing having ceased, the cavity was thoroughly cleansed, the intestines with the omentum carefully replaced, a glass drainage tube (as a precaution) placed in the lower part of the abdominal wound, and the wound then brought evenly together and secured with long steel needles, the peritoneum included. The cutaneous surfaces were brought together and secured by interrupted silver wire sutures.

The tumor was found on examination to measure twenty-eight inches in circumference, twelve inches in length and ten inches in diameter, and weighed twenty pounds. She weighed a few weeks before the operation, one hundred and sixty-five pounds; six weeks after, one hundred and twenty-seven pounds. The cautery was not used, happily there being no necessity for it. The wound was then dressed according to Lister's antiseptic method, and the patient removed to bed. The operation lasted two hours and twenty minutes. Bottles of hot water were then applied to her feet, and on each side until she became warm. There was comparatively little shock, the loss of blood being very small in amount. On her removal to bed she was given brandy until she had thoroughly reacted, after which one-fourth grain morphia was given by hypodermic injection.

About one o'clock at night she commenced to vomit, and continued to do so for twenty-four hours almost incessantly. Everything taken she would throw up instantly, at each time vomiting almost *pure bile.* This bilious vomiting I noted as a very unfavorable symptom. Different remedies were tried, such as lime water and milk, hydrarg. sub. mur. in one-half grain doses every half hour, oxalate of cerium, etc., all without any effect; I finally concluded to give *nothing at all* by the mouth, resorting to ice-cold applications to the face, head and chest, also a spray of cologne which was very refreshing, her fever at the time running pretty high. In this manner I succeeded in checking the vomiting, afterwards having no further trouble.

On the morning of the 28th of May the dressings were removed by Dr. M. Regensburger, who had charge of the Lister arrangements. The wound looked well, a slight serous discharge flowing from the drainage tube. The discharge from the uterine wound was greater, and of a sanquino-serous character. There was very little tympanites, and her condition was good. Beef tea and champagne (iced) were given during the following forty-five hours. The abdominal wound was dressed every twenty-four hours, under full spray of carbolic acid (five per cent. solution). The wound was by this time suppurating very freely. The vaginal wound also discharged freely a thick, dark colored and highly offensive pus. This was injected every three hours with two and one-half per cent. solution carbolic acid. No symptoms of peritonitis occurred. Pulse 120, temperature 102° Fahr.

On the morning of the fourth day she asked to read the morning paper. I will not go into a detailed account of every day's treatment, the symptoms, pulse, temperature, etc., according to my notes, as it will consume unncessary time and space. Suffice it to say, at no time did her temperature go beyond 103°, or her pulse higher than 130. I may remark here, that for several weeks before the operation (after she began to break down) her pulse ranged as high as 130, and respiration was very much embarrassed. I regret that I did not take her temperature or note her respirations exactly. The average temperature after the operation was about 101½°, pulse 120. Her diet was light and nutritious. About the fifteenth of June her condition was so good that I neglected to take any further notes of the case, she having only the usual amount of traumatic and suppurative fever. What is very remarkable, she has not suffered any pain from either of her wounds since the operation, whereas, before, her suffering was excruciating, it being necessary to keep her under the influence of morphia all the time for several days before the operation.

I omitted to say the drainage tube, together with the sutures, were removed on the fourth of June. There was no discharge through the tube at all, the drainage being through the vaginal ligatures, or rather the uterine ligatures, the suppuration being very free through this wound. Injections of two and one-half per cent. solution carbolic acid were used three times a day. Tonics of dialyzed iron, calisaya bark elixir, malt extracts, etc., were given three times a day.

July 1. Out of bed, walking about the room *convalescent*; abdominal wound entirely healed; uterine wound still discharging, though not copiously.

July 7. The right half of ligature (uterine) becoming loose, was removed by Dr. Lane; the left still remaining.

July 20. She is now *entirely well.*

* * * Note. The five per cent. solution used in the spray was really about two and one-half per cent., as in mixing with the hot steam from the atomizer it was diluted to that degree of strength.

